# Is there an association between thyroid function abnormalities and breast cancer?

**DOI:** 10.1590/2359-3997000000191

**Published:** 2016-08-23

**Authors:** Anna Angelousi, Evanthia Diamanti-Kandarakis, Evangelia Zapanti, Afroditi Nonni, Eftuxios Ktenas, Aimilia Mantzou, Konstantinos Kontzoglou, Grigorios Kouraklis

**Affiliations:** 1 Department of Internal Medicine Laiko General Hospital National and Kapodistrian University of Athens Greece Department of Internal Medicine, Laiko General Hospital, National and Kapodistrian University of Athens, Greece; 2 Department of Internal Medicine Medical School National and Kapodistrian University of Athens Greece Department of Internal Medicine, Medical School, National and Kapodistrian University of Athens, Greece; 3 Department of Endocrinology and Diabetes Alexandra Hospital Athens Greece Department of Endocrinology and Diabetes, Alexandra Hospital, Athens, Greece; 4 Medical School Laiko Hospital National and Kapodistrian University of Athens Greece Department of Pathology, Medical School, Laiko Hospital, National and Kapodistrian University of Athens, Greece; 5 National School of Public health National and Kapodistrian University of Athens Greece National School of Public health, National and Kapodistrian University of Athens, Greece; 6 Endocrinology laboratory Evgenidion hospital Athens Greece Endocrinology laboratory of Evgenidion hospital, Athens, Greece; 7 Department of Propaedeutic Surgery Laiko General Hospital University of Athens Medical School Greece Department of Propaedeutic Surgery, Laiko General Hospital, University of Athens Medical School, Greece; 8 Department of Propaedeutic Surgery Laiko General Hospital University of Athens Medical School Greece Department of Propaedeutic Surgery, Laiko General Hospital, University of Athens Medical School, Greece

**Keywords:** Breast cancer, hypothyroidism, hyperthyroidism, thyroid antibodies

## Abstract

**Objective:**

The aim of this study was to evaluate the association between thyroid function abnormalities and breast cancer and, in particular, the prognostic markers of breast cancer..

**Subjects and methods:**

Baseline levels of thyrotropin, free triiodothyronine, free thyroxine and thyroid autoantibodies were measured in 97 women with primary breast cancer, 27 women with benign breast disease, and 4 women with atypical ductal hyperplasia. Their baseline levels were compared with those in 48 healthy women with a normal mammography in the last 2 years.

**Results:**

There were no significant associations between history of thyroid disease and breast cancer (p = 0.33). The mean baseline levels of triiodothyronine and thyrotropin did not differ significantly between the compared groups. The mean baseline levels of free thyroxine were found to be significantly higher in the breast cancer group, even after adjusting for thyroid replacement therapy. The presence of thyroid antibodies did not differ significantly between the compared groups. In a subgroup analysis, breast cancer cases with thyroid disease and particularly hypothyroidism had a significantly lower incidence of lymph node metastases compared with breast cancer cases without thyroid disease.

**Conclusions:**

Our data confirmed the proliferative effect of thyroid hormones on breast cells, which had previously been shown in vitro. Additionally, thyroid disease and particularly hypothyroid function appeared to be associated with a lower incidence of lymph node metastases. Further studies to determine the prognostic role of thyroid hormones in breast cancer are warranted.

## INTRODUCTION

Thyroid hormones are involved in regulating mammalian development, cellular differentiation and metabolism ( 1 ). High affinity binding sites for 3-3-5 triiodothyronine (T3) have been identified in nuclei isolated from human tumors, including those in breast cancer (BC), suggesting that thyroid hormones may play a role in the development of BC at the cellular level ( 2 ).

Recent studies in human breast cancer cells lines (MCF-7 and MDA-MB-231) have shown proliferative effects of thyroid hormones on breast tissue through the same signal cascade utilized by estrogens involving phosphoinositol 3-kinase (PI3K) and the mitogen-activated protein kinase (MAPK) ( 3 ). In estrogen receptor positive (ER+) cell lines (MCF-7), T3 mimics estradiol via its binding to ER and induces the expression of progesterone receptor (PR) and growth factor-alpha (TGF-α) mRNAs ( 3 ). In other human cell lines, T3 has been shown to have a mitogenic effect by increasing the expression of the epithelial growth factor receptor (EGF-r) gene ( 4 ). In mammary cells, T3 can also activate the estrogen response element (ERE) ( 5 ), which is necessary for gene transcription and cell proliferation. Similarly, thyroxine (T4), much like estradiol, can activate the MAPK pathway and thus promote cell proliferation ( 6 ). Additionally, thyroid stimulating hormone (TSH) has been shown to have a direct effect on mammary cells. Recently, TSH receptors have been identified in BC tissue with the use of RT-PCR and were correlated to a lower grade of BC ( 7 ).

The potential correlation between thyroid disease and BC was based on the existence of common pathophysiological mechanisms in both glands. Thyroid hyperoxidase (TPO) and lactoperoxidase are immunologically similar enzymes that have been identified in both thyroid and breast tissue ( 8 , 9 ). Sodium-iodide symporter (NIS) ( 10 ) is an intrinsic plasma membrane glucoprotein of thyroid follicular cells that is also expressed physiologically in lactating breast tissue ( 11 ) as well as in BC in some cases ( 12 , 13 ). Despite this pre-clinical evidence, there is a lack of robust clinical evidence on the association between BC and thyroid function abnormalities. The aim of this single-center case control study was to evaluate the association between thyroid function abnormalities and BC and, in particular, the prognostic markers of BC.

## SUBJECTS AND METHODS

### Patient characteristics

One hundred thirty-three women with mammographic abnormalities were evaluated in the 2^nd^ Surgical Department of the University of Athens (Greece) between June 2010 and March 2014; the women were evaluated before receiving any surgery or medical treatment (chemo and/or radiotherapy). All patients underwent fine needle aspiration of suspicious lesions followed by surgery. Three patients were excluded because of recurrence of known BC, and 2 patients were excluded because of a histological diagnosis of melanoma and sarcoma.

Our cohort consisted of 97 cases of primary BC, 27 cases of benign breast disease (BBD), primarily fibroadenomas and fibrocystic disease, and 4 cases of atypical ductal hyperplasia. Seventy-eight of the 97 cases with BC were diagnosed with ductal breast cancer; 75 cases (77.3%) were invasive ductal breast cancer, 3 (3.1%) were ductal *in situ* cancer (DCIS), and 19 (19.6%) were lobular infiltrating breast cancer. BC tumors were classified according to the Nottingham grading system and the American Joint Committee on Cancer stages. Fifty-nine (61%) of the 97 BC cases were grade I, 28 cases (29%) were grade II, and 10 cases (10%) were grade III. Three (3%) cases had stage 0 tumors ( *in situ* ), 44 (45.4%) had stage I, 29 (30%) had stage II, and 21 (21.6%) had stage III tumors. Seventy-five cases (77.6%) with primary BC showed positive ER and PR.

The control group consisted of 48 non-hospitalized healthy women who were recruited from the family members of the patients in the geriatric clinic of our hospital. All women were from the same geographical area as the cases, and they were required to have had a normal mammogram within the past 2 years.

Women (cases and controls) with a personal history of a previous cancer were excluded from the study. History of thyroid disease was retrieved from the patient’s medical records and/or patient history.

Written informed consent was obtained for each patient and control. The protocol of the study was reviewed and approved by the scientific board of the institutional research committee (Laiko hospital, University of Athens) and was in accordance with the international ethical standards (Declaration of Helsinski).

### Laboratory measurements

Thyroid function tests were performed before surgery or treatment for breast cancer. TSH (normal range: 0.3-4.0 mU/L) was measured with the double enzyme radioimmunoassay (RIA) ‘sandwich’ method (TSH-DiaSorin – CTK-3 Kit, Italy). Free thyroxine (fT4) (normal range: 10-25 pmol/L) and free triiodothyronine (fT3) (normal range: 2.3-5.3 pg/mL) were measured in serum using the double enzyme radioimmunoassay ‘sandwich’ method (ThermoScientific kit, USA). Autoantibodies against thyroglobulin (Tg-Abs) (positivity > 10 mU/L) and thyroid peroxidase (TPO-Abs) (positivity > 50 mU/L) were measured with RIA (ThermoScientific kit, USA). All TSH, fT3, fT4, TPO-Abs and Tg-Abs measurements were performed in the biochemical laboratory of the ‘Laiko’ hospital in Athens, Greece. The TSH receptor antibodies (TRAK) (positivity > 2 U/L) were measured with the receptor radioassay (RRA) method (Thermo Fisher-BRAHMS 101.5 kit, Germany) in the endocrinology laboratory of Evgenidion hospital in Athens, Greece.

### Immunohistochemistry

ER and PR expression were immunohistochemically detected with the commercially available ER (clone EP1) and PR antibodies (clone PgR636) (DAKO), according to ASCO recommendations ( 14 ).

### Statistical analysis

Continuous variables were initially evaluated with the Kolmogorov-Smirnov test. Variables with a normal distribution were evaluated using ANOVA. Variables without a normal distribution (values of fT3, fT4, TSH, TPO-Abs, Tg-Abs, and TRAK) were analyzed using the Kruskall-Wallis test. The categorical parameters were evaluated with Chi-Square test. The results were considered significant at p < 0.05 (95% CI). Cases with atypical ductal hyperplasia (n = 4) were excluded from the statistical analysis because of their small sample size. Statistical analyses were performed using SPSS software (version 20).

## RESULTS

The clinical and epidemiological characteristics of the participants are summarized in Table 1 . The 3 groups (controls, BC cases and BBD cases) differed significantly in mean age, age of menarche, menopausal status and alcohol use.

**Table 1:** Epidemiological characteristics of the studied population

Groups/Epidemiological data	Controls	BC	BBD	Atypical hyperplasia	Statistical significance (p)
Total number	48	97	27	4	
Age (mean ± SD)	54.27 ± 12.84	64.02 ± 14.11	52.07 ± 16.93	60.31 ± 13.86	p < 0.05^1^
BMI (kg/m^2^)	27.02 ± 7.53	28.00 ± 7.09	25.60 ± 5.30	27.42 ± 0.00	p = 0.226^1^
Age of menarche (mean ± SD)	13.43 ± 1.55	11.85 ± 3.07	12.26 ± 3.09	12.39 ± 2.75	p = 0.002^1^
Menopausal status					
Premenopausal (%)	17/48 (35%)	18/90 (20%)	13/24 (54%)	2/4	p = 0.044^1^ (between pre and post-menopausal)
Postmenopausal (%)	30/48 (62.5%)	68/90 (76%)	10/24 (42%)	2/4	
Perimenopausal (%)	1/48 (2%)	4/90 (4%)	1/24 (4%)	0	
History of pregnancy					
Yes (%)	37/48 (78%)	80/91 (88%)	17/24 (71%)	¾	p = 0.44^1^
No (%)	11/48 (22%)	11/91 (12%)	7/24 (29%)	¼	
Parity					
1-2	34/37 (92%)	65/78 (83%)	10/17 (65%)		
> 3	3/37 (8%)	15/78 (17%)	4/17 (35%)		
Age of 1^st^ birth					
< 20	6	8	4	0	p = 0.46^1^
21-30	11	25	6	0	
> 31	10	21	2	0	
Use of contraceptives					
Yes (%)	4/39 (10%)	8/79 (10%)	5/16 (31%)	0/2	p = 0.11^1^
No (%)	35/39 (90%)	71/79 (90%)	11/16 (69%)	2/2	
Smoking					
Yes (%)	12/48 (25%)	22/88 (25%)	6/25 (24%)	¼	p = 1.0^1^
No (%)	36/48 (75%)	66/88 (75%)	19/25 (76%)	¾	
Alcohol					
Yes (%)	1/48 (2%)	5/83 (6%)	5/24 (21%)	0/4	p = 0.025^1^
No (%)	47/48 (98%)	78/83 (94%)	19/24 (79%)	4/4	
1^st^ degree family history of BC					
Yes	7/48 (15%)	14/93 (15%)	4/24 (16.6)	¼	p = 0.95^1^
No	41/48 (85%)	79/93 (85%)	20/24 (83.3)	¾	

^1^ Statistical significance after comparison of controls, BC and BBD group (atypical hyperplasia group was excluding).

BC: breast cancer; BBD: breast benign disease; SD: standard deviation; BMI: body mass index.

### Association between history of thyroid disease and histopathological parameters of breast tissue

The overall frequency of self-reported thyroid disease was 32 of 97 (33%) BC cases, 8 of 27 (29.6%) BBD cases, 3 of 4 (75%) patients in the atypical ductal hyperplasia group and 22 of 48 (45.8%) controls. History of thyroid disease did not significantly differ between the compared groups (p = 0.33) ( Table 2 ). Autoimmune thyroid disease (Hashimoto’s) and goiter were the most frequent thyroid diseases in the studied groups. Hashimoto’s disease was more frequent in the controls than in the BC cases, whereas goiter was more frequent in BC cases than in controls; however, a statistical analysis was not possible because of the small sample size.

**Table 2:** Frequency of thyroid disorders and thyroid treatment in controls, BC and BBD cases

Groups/Frequency of thyroid disorder and treatment	Controls	BC	BBD	Atypical hyperplasia	Statistical significance (p)
History of thyroid disorders					
Yes (%)	22/48 (46%)	32/97 (33%)	8/27 (30%)	3/4	p = 0.33^1^
No (%)	26/48 (54%)	62/97 (64%)	17/27 (63%)	1/4	
No data	0	3/97 (3%)	2/27 (7%)	0	
Type of thyroid disorder					
Hashimoto (%)	7/22 (32%)	9/32 (28%)	3/8 (37.5%)	0	nd^2^
Primary hypothyroidism (other causes) (%)	0	4/32 (12.5%)	0	2	
Hyperthyroidism (%)	4/22 (18%)	4/32 (13%)	0	0	
Goiter (%)	2/22 (9%)	9/32 (28%)	5/8 (62.5%)	1	
Nodules (%)	9/22 (40%)	5/32 (16%)	0	0	
No data	0	1/32 (3%)	0	0	
Treatment					
Yes (substitution, antithyroid drugs)	7/47 (15%)*	21/97 (22%) 3/97 (3%)	7/27 (26%)	3/4	p = 0.79^1^
No	40/47 (85%)	73/97 (75%)	20/27 (74%)	1/4	

* One patient with hyperthyroidism at the past had received I^131^. ^1^ Statistical analysis excluded the atypical hyperplasia group. ^2^ Statistical analysis not possible because of the small sample. BC: breast cancer; BBD: breast benign disease; nd: no data.

Twenty-one of the 97 patients in the BC group (22%), 7 of 27 in the BBD group (26%) and 7 of 47 (15%) in the control group reported levothyroxine treatment at the time of screening. The statistical analysis found no significant differences concerning levothyroxine treatment between the compared groups (p = 0.79). Three cases reported use of antithyroid drugs in the past, and one case reported treatment with iodide (I^131^).

### Association between thyroid function tests and histopathological parameters of breast tissue

The mean baseline values of TSH were 2.37 ± 2.3, 2.07 ± 1.04, 1.88 ± 1.07 and 2.05 ± 1.6 mU/L in the BC, BBD, atypical ductal hyperplasia and controls groups, respectively. Abnormally high levels of TSH (> 4.0 mU/L) were recorded in 15.3% of the BC cases and in 5% of the BBD cases. None of the controls had abnormally high levels of TSH. Abnormally low levels of TSH (< 0.3 mU/L) were recorded in 2.6% of the BC cases and in 2% of the controls. None of the BBD patients had abnormally low levels of TSH. No significant association was found between the mean levels of TSH (p = 0.757) of the compared groups, although BC cases showed slightly higher mean levels of TSH than the other groups ( Table 3 ).

**Table 3:** Mean baseline levels of thyroid hormones (fT3, fT4) and of TSH in controls, BC and BBD cases

Groups/Mean values ± SD, (n)	Controls	BC	BBD	Atypical hyperplasia	Statistical significance (p)
TSH (0,3-4,0 mU/L)	2.05 ± 1.57 (n = 42)	2.37 ± 2.30 (n = 78)	2.07 ± 1.03 (n = 20)	1.88 ± 1.90 (n = 4)	p = 0.757^1^
fT3 (2,3-5,3 pg/mL)	3.47 ± 1.93 (n = 41)	3.20 ± 0.74 (n = 69)	3.11 ± 0.83 (n = 16)	3.33 ± 0.37 (n = 3)	p = 0.622^1^
fT4 (10-25 pmol/L)	15.50 ± 3.76 (n = 41)	17.20 ± 3.30 (n = 67)	16.97 ± 3.29 (n = 16)	18.38 ± 2.80 (n = 4)	p = 0.035^1^

^1^ Analysis was performed with the use of Kruskal-Wallis test. Cases with atypical hyperplasia were excluded from the analysis.

BC: breast cancer; BBD: breast benign disease; SD: standard deviation; TSH: thyroid stimulating hormone; fT3: free triiodothyronine; fT4: free thyroxine.

The mean baseline values of fT3 were 3.18 ± 0.74, 3.11 ± 0.83, 3.33 ± 0.37 and 3.47 ± 1.93 pg/mL in the BC, BBD, atypical ductal hyperplasia and controls groups, respectively. Abnormally low levels (< 2.3 pg/mL) of fT3 were recorded in 10% of the BC cases, 6% of the BBD cases and in 12% of the controls. Abnormally higher levels of fT3 (> 5.3 pg/mL) were recorded in only 2% of the controls. None of the cases (BC and BBD) had abnormally higher levels of fT3. No significant association was found between the compared groups’ mean values of fT3 (p = 0.622) ( Table 3 ).

The mean baseline values of fT4 were 17.2 ± 3.28, 16.9 ± 3.29, 18.38 ± 2.8 and 15.5 ± 3.76 pmol/L in the BC, BBD, atypical ductal hyperplasia and control groups, respectively. All BC cases and controls had normal levels of fT4. The mean values of fT4 were significantly higher in the BC group than in the other groups (p = 0.035). This statistical significance, although attenuated (p = 0.055), remained present when patients with levothyroxine treatment were excluded.

### Association between thyroid autoantibodies and histopathological parameters of breast tissue

The frequency of positive TPO-Abs was 21.4% in the BC group, 33.3% in the BBD group, and 18% in controls. Positive Tg-Abs were found in 65% of the cases in the BC group, 55.5% in the BBD group and 16.6% in the control group. The levels of TPO-Abs and Tg-Abs did not significantly differ between the compared groups (p = 0.43 for TPO-Abs and p = 1.00 for Tg-Abs) ( Table 4 ). The tests for TRAK antibodies were negative for the whole study population (cases and controls) (positivity > 2 U/L). The mean values of TRAK did not differ significantly (p = 0.42) between the groups.

**Table 4:** Frequency and mean baseline values of thyroid autoantibodies in the compared groups

Groups/Frequency and mean values ± SD, (n)	Controls	BC	BBD	Atypical hyperplasia	Statistical significance
**TPO-Abs**					

Μean values ± SD (n)	149.50 ± 495.54 (n = 39)	312 ± 810 (n = 42)	520 ± 1089 (n = 12)	20 ± 26.54 (n = 2)	p = 0.6^1^
Negative (< 50 mU/L)	32/39 (82%)	33/42 (78.5)	8/12 (66.6%)	2/2	p = 0.43^1^
Positive (> 50 mU/L)	7/39 (18%)	9/42 (21.4%)	4/12 (33.3%)	0/2	

**Tg-Abs**					

Mean values ± SD (n)	28.48 ± 64 (n = 12)	132 ± 238 (n = 17)	17.41 ± 14.65 (n = 9)	nd	p = 0.07^1^
Negative (< 10 mU/L)	10/12 (83%)	6/17 (35%)	4/9 (44.4%)	nd	p = 1.00
Positive (> 10 mU/L)	2/12 (16.6%)	11/17 (64.7%)	5/9 (55.5%)	nd	

**TRAK**					

Mean values ± SD (n)	0.71 ± 0.23 (n = 41)	0.64 ± 0.12 (n = 20)	0.66 ± 0.20 (n = 3)	nd	p = 0.42^1^
Negative (> 2 U/L)	41/41	20/20	3/3	nd	
Positive (< 2 U/L)	0	0	0	nd	

^1^ Atypical hyperplasia was excluded from the statistical analysis. Analysis was performed with the use of Kruskal-Wallis test.

BC: breast cancer; BBD: breast benign disease; SD: standard deviation; nd: no data; TPO-Abs: thyroid hyperoxidase antibodies; TgAbs: thyroglobulin antibodies; TRAK: TSH receptors antibodies.

### Association between thyroid function and hormone dependent BC (ER and/or PR positivity)

Fifty-nine BC cases out of a total of 76 (77.6%) were found positive for estrogen and/or progesterone receptors through immunohistochemistry analysis ( Table 5 ). Statistical analysis found no significant differences in the prevalence of history of thyroid disease between the hormone-dependent and hormone-independent BC cases (p = 0.47). Further analyses (not shown in Table 5 ) did not show any significant difference between hormone-dependent BC and positive thyroid autoantibodies (7 out of 25 hormone-dependent BC cases were positive for TPO-Abs, p = 0.15 and 6 of 11 hormone-dependent BC cases had positive Tg-Abs, p = 1.00) nor between the mean levels of thyroid hormones (p = 0.486 for TSH levels, p = 0.229 for fT3 levels and p = 0.15 for fT4 levels) and hormone-dependent BC.

**Table 5:** Frequency of history of thyroid disease in the differenent T, N stages, the different grades (I, II, III) and the ER/PR positive BC

Thyroid disease/Prognostic factors of BC	History of thyroid disease		Statistical significance (p)

	Yes	No	
TNM stage			
pT1	14 (14.4%)	27 (28%)	p = 0.805*
pT2	12 (12.4%)	24 (25%)	
pT3	4 (4%)	8 (8.2%)	
pT4	1 (1%)	2 (2%)	
pT+	8 (23.5%)	26 (76.5%)	p = 0.034
pT-	21 (46.6%)	24 (53.3%)	
Grade			
I	2 (25%)	6 (75%)	p = 0.83
II	17 (33%)	35 (66%)	
III	7 (22%)	18 (78%)	
Hormone-sensitivity			
ER +/PR +	20 (34%)	39 (66%)	p = 0.47
ER -/PR-	5 (33.5%)	10 (66.6%)	

* Cases with T4 tumors were excluded because of the small sample size.

BC: breast cancer; BBD: breast benign disease; ER: estrogen receptors; PR: progesterone receptors.

### Association between thyroid disease and tumor size, nodal involvement or grading of BC

BC cases with a self-reported history of thyroid disease presented less frequently with lymph node infiltration (pN) than the BC cases without history of thyroid disease (p = 0.035) ( Table 5 ). The frequency of primary hypothyroidism in BC cases with lymph node infiltration was 37.8% (2 patients with Hashimoto’s and 1 with primary hypothyroidism out of 8 cases) and was 47.6% (7 patients with Hashimoto’s and 3 with primary hypothyroidism out of 21 cases) in BC cases without lymph node infiltration.

BC cases with tumors with lymph node infiltration presented lower mean TSH levels and higher fT4 levels (mean value of TSH = 1.97 ± 1.4 mU/L, of fT3 = 3.37 ± 0.78 pg/mL and of fT4 = 17.68 ± 3.16 pmol/L) than the tumors without lymph node infiltration (mean value of TSH = 3.99 ± 1.0 mU/L, of fT3 = 3.09 ± 0.66 pg/mL and of fT4 = 16.33 ± 3.5 pmol/L), although there were no significant differences (p = 0.148 for TSH, p = 0.26 for fT3 and p = 0.113 for fT4). Due to the small sample of the T4 stage (n = 3), the corresponding statistical analyses were not performed.

The presence of positive thyroid autoantibodies was not significantly associated with the pT stage of BC (p = 0.31 for TPO-Ab and p = 1.0 for Tg-Ab), the pN stage of BC (p = 0.87 for TPO-Ab and p = 0.76 for Tg-Ab), nor the grade of BC (p = 0.803 for TPO-Ab and p = 0.348 for Tg-Ab ( Table 5 ).

## DISCUSSION

The present study showed that the mean baseline levels of fT4 were significantly higher in BC cases than in controls and patients with BBD. Additionally, history of thyroid disease was associated with a significantly lower frequency of lymph node metastases in BC cases. Recent data have shown that BC remains one of the most common cancers worldwide, with an estimated incidence of 464,000 new BC cases per 3.45 million new cases of cancer in Europe ( 15 ).

Previous studies are consistent with our results, showing no significant association between history of thyroid disease and histopathological characteristics of breast diseases ( 1 , 16 - 20 ). Nevertheless, other studies have demonstrated a higher ( 8 , 21 , 22 ) or a lower incidence of thyroid disease in BC cases ( 23 ). Hashimoto’s disease, with or without positive thyroid antibodies (TPO-Abs and Tg-Abs), has been shown to be associated with an increased risk of BC ( 8 , 9 , 24 - 27 ) and, according to some studies, with a better prognosis and increased disease-free survival in BC cases ( 16 , 28 ). In our study, it seemed that Hashimoto’s disease was not associated with BC; however, the small sample size precluded any definitive conclusions.

Our data showed that the mean baseline levels of fT4 were significantly higher in BC cases compared with the other groups, which is consistent with other studies in the literature ( 1 , 29 ). This correlation was sustained after exclusion of patients with levothyroxine treatment. Recently, it was found that fT4 levels in women without known thyroid disease and levothyroxine treatment were positively associated with risk of breast cancer, particularly in women with a BMI > 25 ( 28 ). However, hypothyroidism and low levels of fT4 ( 21 ) or only low levels of fT4 with normal TSH have also been shown to be related to an increased risk of BC risk in women ( 29 ).

As with fT4 levels, there have been reports suggesting an association of higher ( 1 , 30 ) or lower levels of fT3 with risk of BC ( 29 , 31 , 32 ). Surprisingly, we found no significant association between the mean baseline levels of fT3 and BC. One possible explanation for this result could be the short half-life of fT3 compared to fT4, which makes fT3 a less reliable marker of thyroid function. Another potential reason could be the circadian rhythm and periodicity concerning TSH and fT3 levels in particular. This circadian variation is not as evident in fT4 levels ( 33 ).

The mean baseline levels of TSH were not found to be significantly different between groups. Although there have been data associating low baseline levels of TSH (< 0.5 mU/l) in patients without known thyroid disease or treatment with a significantly increased risk of lung and prostate cancer, this association in BC has not been clearly demonstrated ( 34 ).

The association between positive thyroid autoantibodies and BC has been largely discussed in the literature ( 35 , 36 ). TPO-Abs are not thyroid-specific enzymes, and they can also be expressed in other tissues ( 24 ). In particular, TPO mRNA and protein have been found to be weakly but clearly expressed in BC tissues, peri-tumor tissues and in fat depots, and although they lack their functional activity, they maintain their antigenic role and can trigger B lymphocytes ( 37 ). Clinically, TPO-Abs have been associated with both an increased risk of BC ( 8 , 9 , 38 ) and a lower risk of BC; in some cases, TPO-Abs have even been associated with a lower frequency of distant metastases ( 39 ), a better outcome and better overall survival **(** 27,38). Our data did not find an association between thyroid autoantibodies and risk of BC, which is consistent with a prospective study by Kuijpens and cols. ( 21 ).

Few data exist regarding the role of Tg-Abs and TRAB in BC. Tg-Abs have been associated with different oncological diseases and have been found to be more prevalent in patients with BC ( 23 ); however, they are not specific and may be influenced by different environmental factors ( 40 ). Extrathyroidal expression of TSH receptor has also been demonstrated in cardiac muscle and adrenal and kidney tissue ( 41 ) and was recently confirmed in BC tissue samples using RT-PCR ( 7 ). In clinical studies, TRAB levels have been found to be significantly higher in BC cases, particularly in low-grade BC cases ( 7 ), compared with healthy controls ( 40 ) and BBD cases ( 1 ).

The data in the literature associating thyroid function with prognostic markers of BC are scarce. In one study, BC cases with primary hypothyroidism showed a more indolent invasive breast cancer with less frequent lymph node metastases compared with euthyroid BC cases ( 23 ). According to other data, TPO-Ab levels ( 27 ) as well as the presence of nuclear TR-α receptors in breast cancer cells *in vitro* ( 2 , 41 ) were inversely correlated with lymph nodes metastases. Our findings showed that a history of thyroid disease was significantly correlated with a lower incidence of lymph nodes (pN stage) metastases in BC cases. The frequency of primary hypothyroidism (mainly Hashimoto’s) was higher in BC cases with no lymph node involvement than in other thyroid diseases; however, the small sample size did not allow for a meaningful statistical analysis. We did not perform analyses for other prognostic markers such as Ki-67 and HER2 because of the limited data.

These data should be analyzed with caution because of the small size sample and because patients with known thyroid disease have more frequent access to hospital facilities and could thus be diagnosed with BC in earlier stages, which would explain the absence of lymph node metastases. Another limitation of this study was that patient’s history of thyroid disease and treatment was based on medical interviews and files, and this could have led to misclassifications in the diagnosis of thyroid disease.

In conclusion, our findings showed that a history of thyroid disease was not associated with risk of BC. However, a history of thyroid disease was associated with a significantly lower frequency of lymph node metastases, suggesting the potential for a better prognosis in these BC cases. The significantly higher mean baseline levels of fT4 in the BC cases compared with the other two groups may suggest a thyroid hormone pattern that is distinct from that of low T3 syndrome. Further studies to determine the prognostic role of thyroid hormones in BC are warranted, as the small number of cases in the present study precludes definitive conclusions.
